# Draft Genome Sequences of Two Thermophilic Moorella sp. Strains, Isolated from an Acidic Hot Spring in Japan

**DOI:** 10.1128/MRA.00663-19

**Published:** 2019-08-01

**Authors:** Yuto Fukuyama, Ayumi Tanimura, Masao Inoue, Kimiho Omae, Takashi Yoshida, Yoshihiko Sako

**Affiliations:** a Laboratory of Marine Microbiology, Graduate School of Agriculture, Kyoto University, Kitashirakawa Oiwake, Sakyo, Kyoto, Japan; University of Southern California

## Abstract

The thermophilic Moorella sp. strains E308F and E306M were isolated from an acidic hot spring in Japan. Here, we report the draft genome sequences of E308F (3.06 Mbp; G+C content, 54.0%) and E306M (2.99 Mbp; G+C content, 54.4%), to advance the genomic information available on the genus *Moorella*.

## ANNOUNCEMENT

The genus Moorella is often represented by anaerobic, thermophilic, and CO-utilizing bacteria that use the Wood-Ljungdahl pathway for CO or CO_2_ fixation and acetogenesis (1). Recently, some strains and species of *Moorella* were reported as hydrogenogenic CO-oxidizing bacteria (2, 3). Here, we successfully isolated two novel *Moorella* sp. strains, E308F and E306M, under 100% CO cultivation, and we report their draft genome sequences.

Samples were collected from Unagi-onsen (31°13’39”N, 130°36’46”E), an acidic hot spring in Japan. For isolation, the cells were grown at 65°C on modified hypotonic artificial seawater (hASW) medium (4) under 100% CO. Modifications of this medium were as follows: the concentration of yeast extract was increased to 1.0 g/liter, and sodium pyruvate (1.0 g/liter) was supplemented. To identify the isolates (strains E308F and E306M), we extracted their genomic DNA using a DNeasy blood and tissue kit (Qiagen, Valencia, CA, USA). Their partial 16S rRNA genes were amplified using the universal primers B27f (5′-AGAGTTTGATCCTGGCTCAG-3′) and U533r (5′-TTACCGCGGCKGCTGRCAC-3′) (5, 6) for Sanger sequencing. The sequence data showed the highest sequence identity with the 16S rRNA genes of Moorella humiferrea (96.2% and 97.4% for E308F and E306M, respectively) by BLASTN search (7), suggesting the assignment of the two strains to the genus *Moorella*.

For genome sequencing, the isolates were grown on modified hASW medium. Mate pair libraries were prepared from DNA using a Nextera mate pair library preparation kit (Illumina, Inc., San Diego, CA, USA), followed by sequencing with the Illumina MiSeq platform using a v3 reagent kit (2 × 300-bp mate pair reads). Totals of 4,147,082 and 3,134,908 reads were generated for E308F and E306M, respectively. The generated reads were filtered and trimmed using Trimmomatic v0.36 (8). The Nextera mate pair junction adaptor sequences were trimmed using NxTrim v0.4.1 (9), and only proper mate pair reads (3,796,450 and 2,817,228, respectively) were assembled into scaffolds using SPAdes v3.13.0 (10). After the correction of large-scale misassemblies by examination of the distribution of Nextera mate pair insert sizes using NxRepair v0.13 (11), the assembled scaffolds were analyzed using DFAST server v1.2.0 (12) to predict open reading frames (ORFs) with annotation. The average nucleotide identity (ANI) was calculated by JSpeciesWS (13). All analyses were performed with default parameter settings.

The draft genomes of E308F and E306M were assembled into 10 and 6 scaffolds with 373× and 283× average genome coverages, *N*_50_ sizes of 1,211,500 bp and 2,985,197 bp, total lengths of 3,057,355 bp and 2,985,197 bp, and average G+C contents of 54.0% and 54.4%, containing 3,106 and 3,051 predicted ORFs, respectively. The ANI percentage between the two strains was 98.9%. In contrast, the ANI percentages between strain E308F and other published *Moorella* genomes ranged from 78.5% (Moorella thermoacetica ATCC 39073; GCF_000013105) to 90.1% (Moorella mulderi DSM 14980; GCF_001594015), which were below the proposed 95% cutoff for the genome definition of a species (14).

Both strains possessed two CO dehydrogenase (CODH) genes, and these amino acid sequence alignments showed their conserved active site residues (15, 16). Of these, one CODH gene was flanked by an energy-converting hydrogenase, implying the capability of hydrogenogenic carboxydotrophy (17).

### Data availability.

The genome sequences of *Moorella* sp. strains E308F and E306M have been deposited in the DNA Data Bank of Japan (DDBJ) under accession numbers BJKN01000000 and BJKO01000000, respectively. Sequence data have been deposited in the DDBJ Sequence Read Archive under the accession numbers DRX170528 and DRX170529, respectively.
